# HCl-induced acute lung injury: a study of the curative role of mesenchymal stem/stromal cells and cobalt protoporphyrin

**DOI:** 10.1186/s43141-021-00139-w

**Published:** 2021-03-15

**Authors:** Reham A. EL-Shahat, Reda S. EL-Demerdash, El Said El Sherbini, Entsar A. Saad

**Affiliations:** 1 Chemistry Department, Faculty of Science, Damietta University, Damietta, Egypt; 2 Urology & Nephrology Center, Faculty of Medicine, Mansoura University, Mansoura, Egypt; 3 Biochemistry and Chemistry of Nutrition, Faculty of Veterinary Medicine, Mansoura University, Mansoura, Egypt

**Keywords:** COVID-19, Lung injury, Oxidative stress, Apoptosis, Inflammation

## Abstract

**Background:**

This study was designed to investigate bone marrow mesenchymal stem/stromal cells (BM-MSCs) and cobalt protoporphyrin (CoPP) curable effects on HCl-induced acute lung injury (ALI) and its underlying mechanisms hoping this might aid to offer a therapeutic opportunity for ALI.

**Results:**

Forty male Sprague Dawley rats were randomly allocated into four groups; normal (normal rats), ALI (rats injected with 2 ml hydrochloric acid (HCl)/kg via trachea), ALI + BM-MSCs (ALI rats intravenously injected twice with 1 × 10^6^ BM-MSCs/rat/week), and ALI + CoPP (ALI rats intraperitoneally injected twice with CoPP (0.5 mg/100 g/week)). White blood cells (WBCs), red blood cells (RBCs), hemoglobin (Hb), serum tumor necrosis factor-alpha (TNF-α), lung histopathology, apoptosis markers (*caspase-3* and *Bcl2*), and oxidative stress markers (malondialdehyde (MDA), superoxide dismutase (SOD), and catalase (CAT)) were measured. ALI caused increases in WBCs, TNF-α, *caspase-3*, and MDA, and morphological damage score of lungs with decreases in RBCs, Hb, *Bcl2*, SOD, and CAT (*p* < 0.05). BM-MSCs or CoPP treatment reversed these ALI-induced changes (*p* < 0.05) towards normal.

**Conclusions:**

BM-MSCs and CoPP could attenuate ALI by modulation of inflammation, oxidative stress, and apoptosis. Curative roles of BM-MSCs were more effective than those of CoPP. This highlights BM-MSCs as a potent therapy for HCl-associated ALI.

## Background

Acute lung injury (ALI) is a disease associated with vigorous inflammatory response resulting in deterioration of gas exchange [1], and until this moment, there is no successful treatment for it. Lung edema is one of ALI manifestations that might advance to hypoxemia and may lead finally to acute respiratory distress syndrome (ARDS) [2]. Novel coronavirus disease 2019 (COVID-19) is one of the acute respiratory diseases that initially cause lung damage [3]. In the intensive care unit (ICU), nearly one third (30%) from COVID-19 patients showed development of severe lung edema, dyspnea, hypoxemia, or ARDS. Among ICU and non-ICU COVID-19 patients, nearly 65% of those who developed ARDS died [2]. Thus, ALI represents the most severe form of the viral infection sustained by the pandemic COVID-19 [4] and ARDS, as one of the severe complications of ALI, remains a major cause of morbidity and mortality in critically ill patients with ALI. Since acute pulmonary inflammatory response is a criterion of ALI/ARDS, excessive pulmonary inflammation is involved in ALI/ARDS pathogenesis that finally results in alveolar-capillary barrier impairment and gas exchange deterioration [5]. Therefore, a therapy or an add-on therapy that has potent anti-inflammatory influence in lung injury along with the drugs already clinically used could introduce a better therapeutic outcome for patients.

Gastric aspiration, materials inhalation from the stomach into the airways beyond the vocal cords, frequently arises in patients in the ICU. Depending on the aspirate volume, gastric aspiration has the ability to injure lungs starting from subclinical pneumonitis to advanced respiratory failure. Aspirate content may include particles of food, gastric acid (HCl; hydrochloric acid), blood, or bacteria. Among these contents, HCl has the greatest influence on the lung injury [6].

Stem cells are clonogenic cells that have an exceptional fit of self-renewal and multilineage differentiation. They have been isolated from a great group of tissues [7]. Accordingly, they have manifold applications in tissue engineering, regenerative medicine, cell therapy, and gene therapy as they have a high differentiating capacity [8]. Mesenchymal stem/stromal cells (MSCs) are multipotent cells that are able to form fibroblast-like colonies. MSCs could be isolated from diverse sources like placenta, bone marrow, adipose tissue, etc. Therefore, they offer an attractive cell source for co-transplantation with hematopoietic stem cells and replacement therapy for injured tissues in many patients [9, 10].

Cobalt protoporphyrin (CoPP) is a powerful heme oxygenase-1 (HO-1) inducer. HO-1, a cytoprotective enzyme, catalyzes the rate-limiting step in heme breaking down and degrades the heme into carbon monoxide (CO), biliverdin and iron. HO-1 exhibits a protective action versus oxidative stress. Owing to HO-1 anti-inflammatory and anti-oxidant effects, it is doing a significant part in the maintenance of cellular homeostasis [11].

Therefore, the present study was carried out to isolate adult bone marrow MSCs and to test their ability to treat HCl-induced ALI versus to CoPP in experimental rats. This might aid in offering a therapeutic opportunity for HCl-associated ALI.

## Methods

### Chemical agents

Cobalt protoporphyrin (CoPP) was obtained from Sigma Chemical Company (St. Louis, MO, USA) and dissolved in distilled water in dark tubes. Hydrochloric acid (HCl) was purchased from Sigma Aldrich fine chemicals (Cat #, H1758).

### Animals

Male Sprague Dawley rats (16 weeks old) weighing between 180 and 200 g acquired from Animal House of Nile Center for Experimental Researches, Mansoura, Egypt, were used. They were housed according to the National Institutes of Health guide for the care and use of Laboratory animals (NIH Publications No. 8023, revised 1978) in stainless steel cages in an artificially illuminated and thermally controlled room (22–25 °C and 12-h light/dark cycle). Rats were fed on a normal laboratory rodent diet, and given water *ad libitum* for 1 week of acclimation prior to the experimental work. All animals were received human care in compliance with the guidelines of the Animal Care and Use Committee of Damietta University. The experimental protocol was approved by Chemistry Department, Faculty of Science, Damietta University, Egypt.

### Operation for induction of HCl-induced acute lung injury (ALI)

In brief, under anesthesia (ketamine (75 mg/kg) and xylazine (10 mg/kg)), the rat was placed in a supine position with the extremities pulled caudally to facilitate exposure of the trachea. Then, trachea was exposed through an anterior neck incision and a direct puncture with a 24-gauge needle on a 1-ml tuberculin syringe is performed two to four tracheal rings below the larynx. HCl was injected into the lung in a volume of 2 ml/kg. After instillation of HCl, the tuberculin syringe was removed. The neck was then repaired with sutures. After 7 days, samples from the lungs were taken, and ALI induction was confirmed.

### Isolation and culture of MSCs from bone marrow

**Fig. 1:**
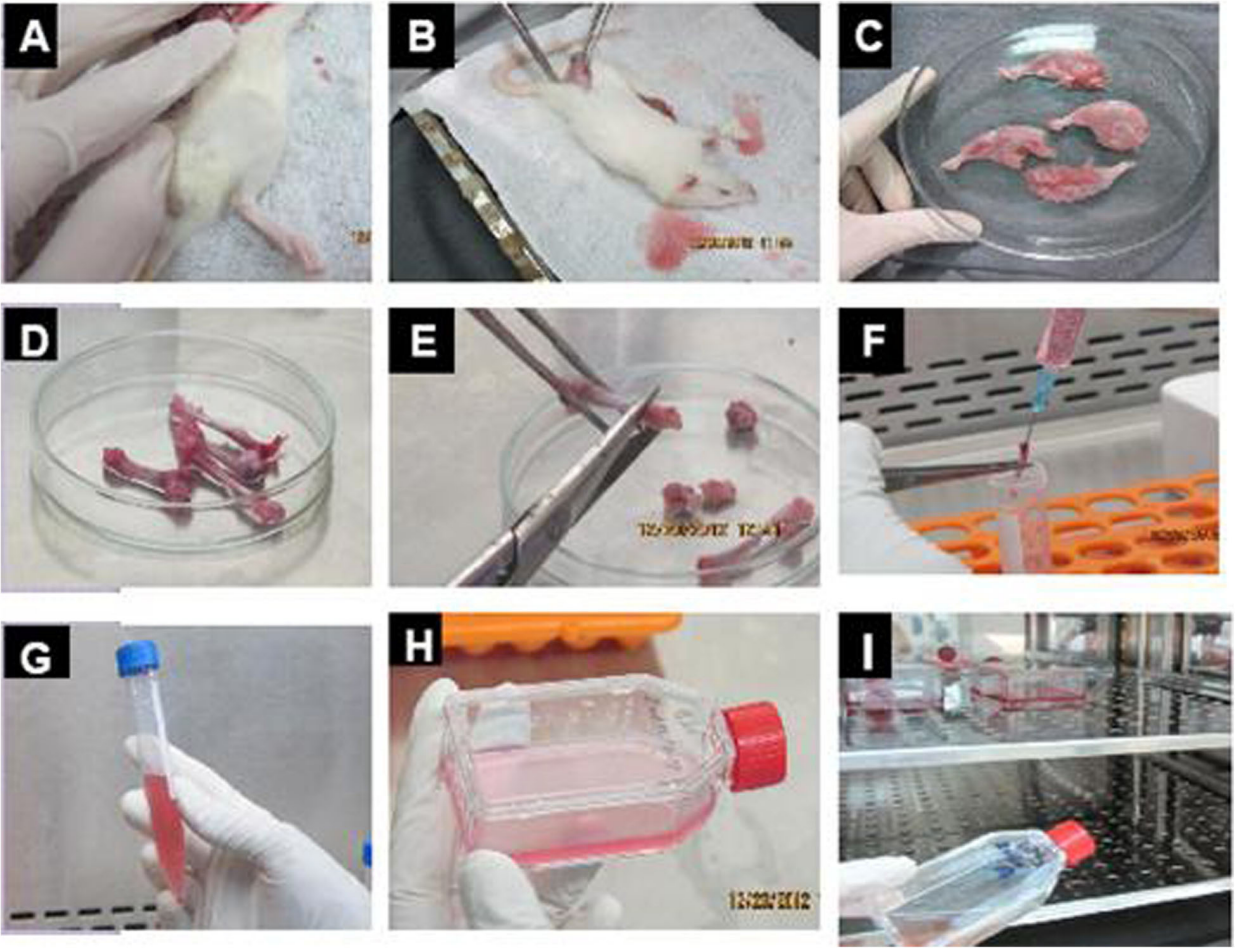
Isolation and culture of bone marrow mesenchymal stem cells (BM-MSCs). **a** Exposure of penile vein. **b** Handling of penile vein. **c** Isolation of bones in petri dish. **d** Cleaning of the bone shaft. **e** Removing of the joints. **f** Flushing of bone marrow. **g** Soaking of the bone marrow by complete media. **h** Transport to the flask. **i** Transport of the flask to CO_2_ incubator

In short, the rats were anesthetized using halothane, then the skin was sterilized by ethyl alcohol 70%, and rats’ femurs and tibia were cautiously excised from adherent soft tissues (Fig. 1a–c). After this, bones were kept in 70% ethyl alcohol for 1–2 min, then dipped in a petri dish containing phosphate-buffered saline (Cat no. BE17-516F, Lonza, USA) for washing. Bone ends were cut by sterile scissors with flushing of the bone marrow with Dulbecco’s modified Eagles medium (DMEM) (Cat no. BE12-719F, Lonza, Belgium) enriched with fetal bovine serum (FBS) 10% (Cat no.10270, Gibco, USA) and 1% penicillin strips (10.000 U penicillin–10.000 μg streptomycin/ml) (Cat no. DE17-602E, Lonza, USA). Cells were seeded in 20 ml complete media and incubated at 37 °C in a 5% humidified CO_2_ incubator (Shel lab, USA) (Fig. 1d–i). One day later, the media were discarded to get rid of the unattached cells. MSCs were differentiated from bone marrow cells by their ability to attach to tissue culture polystyrene flask (75 cm^2^, Greiner Bio-One). Cells were sub-cultured with using 0.25% trypsin/ethylenediamine-tetraacetic acid (Cat no. BE17-161E, Lonza, Belgium).

### Experimental groups

Forty rats were randomly divided into 4 groups of 10 rats in each group:

1. *Group I* (*Control*)*:* served as normal control; aspirate normal saline injected into the lung in a volume of 2 ml/kg (negative control).
2. *Group II* (*ALI*)*:* aspirate HCl (0.1 N, pH 1.25) injected into the lungs in a volume of 2 ml/kg for one time (positive control).
3. *Group III* (*ALI + BM-MSCs*): aspirate HCl, and 1 week later, they were injected intravenously through penile vein with 1 × 10^6^ BM-MSCs/rat for two times; after 1 week of the first dose (1 × 10^6^ BM-MSCs/rat) in 0.2 ml of Dulbecco’s modified Eagles medium (DMEM), rats received the second dose (1 x 10^6^ BM-MSCs/rat) in 0.2 ml DMEM.
4. *Group IV* (*ALI + CoPP*): aspirate HCl (0.1 N, pH 1.25), and 1 week later, they were injected intraperitoneally with CoPP (0.5 mg/100 g of body weight) for two times; after 1 week of the first dose (0.5 mg/100 g of body weight), rats received the second dose (0.5 mg/100 g of body weight).

One week later of the last treatment dose, rats in all groups were sacrificed, and the samples were collected.

### Collection of blood samples and harvesting of lung tissues

At the end of the experiment, all alive rats were sacrificed after anesthesia (ketamine and xylazine), and blood samples were withdrawn in the EDTA-containing tube for blood cell count and in dry tubes to obtain serum. As well, lung tissues were rapidly harvested, and divided into two parts: one part was placed in formalin (10%) for histopathological examination, and the second part was placed in liquid nitrogen for biochemical analyses of markers of oxidative stress, and for molecular study of the expression of *caspase-3* and *Bcl2*.

### Assay of TNF-α in serum

Serum TNF-α was measured via Enzyme-Linked Immunosorbent Assay technique with rat TNF-α kit (eBioscience, Austria) as stated by the instructions of the manufacturer.

### Measurement of oxidative stress markers (MDA, SOD, and CAT)

The lung tissues were excised, washed with 0.9% NaCl solution, and some parts were homogenized for assay of catalase (CAT), malondialdehyde (MDA) and superoxide dismutase (SOD) using their kits (Biodiagnostic, Giza, Egypt), and the assay methods were done according to the manufacturer’s instructions. In brief, for determination of MDA level, thiobarbituric acid was used to react with MDA under acidic conditions at 95 °C for 30 min and the absorbance of the developed pink product was measured at 534 nm. Regarding the colorimetric estimation of SOD activity, the test was based on the ability of SOD to inhibit phenazine methosulfate-mediated reduction of nitro blue tetrazolium dye. While, the test for determination of CAT activity was based on that, each unit of CAT decomposes 1 μM of hydrogen peroxide per minute at 25 °C and pH 7. The reaction was stopped after 60 s using CAT inhibitor, and the remaining hydrogen peroxide gave a colored product by reaction with 3,5-Dichloro-2-hydroxybenzene sulfonic acid and 4-aminophenazone in presence of peroxidase. Color intensity was read at 510 nm, and it was inversely proportional to the amount of CAT in the original sample.

### Real-time (RT)-PCR for *caspase-3* and *Bcl2*

From 30 mg of lung tissues, total RNA was extracted using the RNeasy® Mini Kit (Cat no. 74106). One microgram of the total RNA was reverse transcribed into cDNA with RT2SensiFAST™ cDNA synthesis kit (Cat. No. BIO-65053). RT-PCR with SYBR Green was used to detect the gene expression of *caspase-3* and *Bcl2* using the SensiFAST SYBR® No-ROX Kit (Cat. #BIO-65053). Primer sequences of the tested genes are; *Bcl2*, F: GTACCTGAACCGGCATCT, R: ATCAAACAGAGGTCGCA, *caspase-3*, F: GGCCGACTTCCTGTATGCTT, F: GGCCGACTTCCTGTATGCTT; R: CGTACAGTTTCAGCATGGCG and housekeeping gene *GAPDH*; F: TTGTGCAGTGCCAGCCTCGT, R: TGCCGTTGAACTTGCCGTGG. Gene amplification was carried out with an initial denaturation at 95 °C for 2 min, denaturation at 95 °C for 5 s, annealing at 60 °C for 10 s and extension at 72 °C for 5–20 s, and 40 cycles. By the end of the last cycle, temperature was increased to 95 °C to produce a melt curve. The samples were exposed to PIKOREAL96 Real-time thermal cycler (Thermo Fisher, USA); the relative expressions of the target genes were normalized with *GAPDH*, and calculated by applying the 2^−ΔΔCt^ method.

### Histopathological examination

Ordinarily, fixed lung tissues in 10% neutral buffered formalin were processed into paraffin blocks, 3–5 μm sections were made on slides, stained with hematoxylin and eosin dye, and examined under a microscope, and the degree of fibrosis, necrosis, leucocytic infiltration, and alveolar collapse were scored into four grades: no (-), mild (+), moderate (++), and severe (+++).

### Statistical analysis

The study results were analyzed using the statistical package for social science, version 17 (SPSS Software, SPSS Inc., Chicago, USA), and expressed as means ± standard deviation (SD). For data with Gaussian distribution, statistical analysis was performed using analysis of variance (One-way ANOVA) followed by Tukey’s multiple-comparison test. For parameters with non-Gaussian distribution, Kruskal–Wallis test was employed followed by Dunnett’s test for multiple comparison. Differences considered significant at *p* ˂ 0.05.

## Results

### Morphological characterization of cultured BM-MSCs

**Fig. 2:**
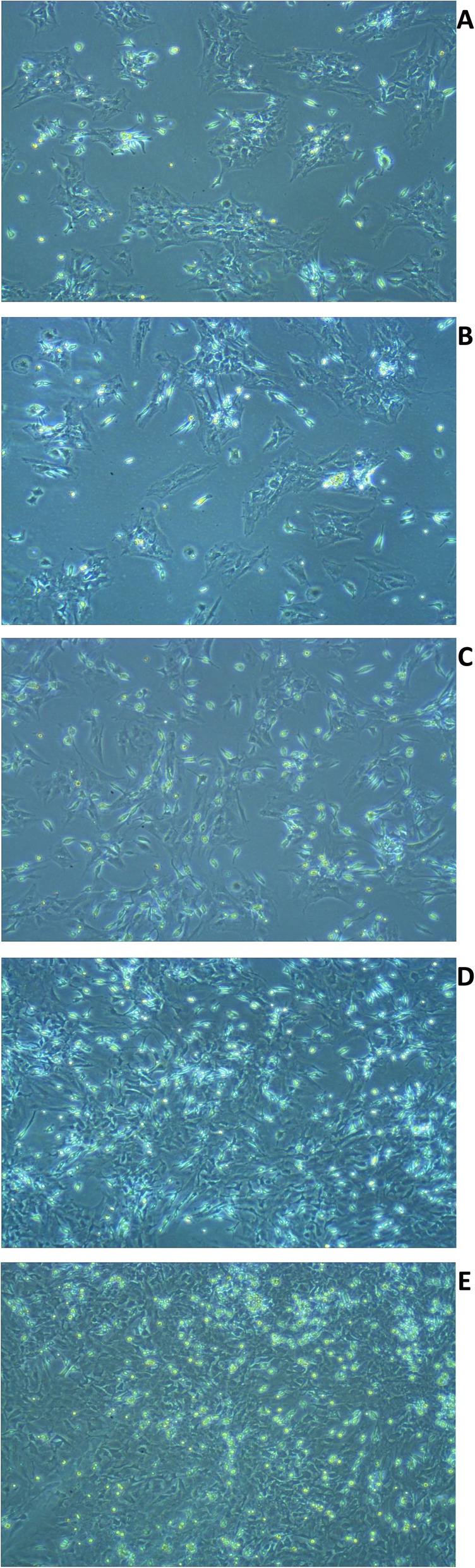
Photomicrograph of bone marrow mesenchymal stem cells (BM-MSCs). **a** Seven days after isolation & culturing showing confluence of 40–50%, **b** 10 days after isolation & culturing showing confluence of 50–60%, **c** 14 days after isolation & culturing showing confluence of 60–70%, **d** 21 days after isolation & culturing showing confluence of 70–80%, and **e** 30 days after isolation & culturing showing confluence of 80–90% (X 100)

The cultured BM-MSCs were photographed to follow-up their growth in culture on days 7, 10, 14, 21, and 30 as illustrated in Fig. 2a–e. With time, the cultured BM-MSCs showed increases in their number indicating high proliferation ability. They seemed as homogenous spindle-shaped fibroblast-like cells. The cells showed confluence of 40–50% on day 7 (Fig. 2a), 50–60% on day 10 (Fig. 2b), 60–70% on day 14 (Fig. 2c), and 70–80% on day 21 (Fig. 2d). BM-MSCs 1 month after isolation showing confluence of 80–90% (Fig. 2e) were harvested and passaged up to passage 4. Cells of passage 3 and passage 4 were used for treatment of rats with HCl-induced ALI in this study.

### Animal survival

**Fig. 3:**
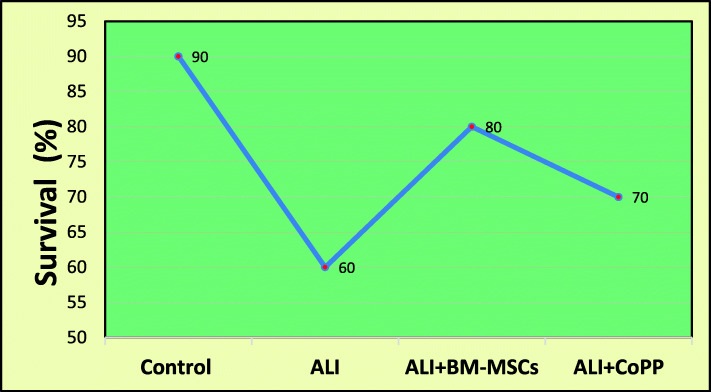
Survival rate in different study groups. *ALI* acute lung injury, *BM-MSCs* bone marrow mesenchymal stem cells, *CoPP* cobalt protoporphyrin

As illustrated in the survival curve (Fig. 3), only one rat (10%) died from the healthy control group due to unknown reason. Four rats (40%) died from the ALI group due to respiratory collapse in the same day of the operation. Two rats (20%) died from the ALI + BM-MSCs group directly after injection with stem cells, and three rats (30%) died from the ALI + CoPP group directly after injection with CoPP.

### Effects of BM-MSCs and CoPP on hematological parameters in HCl-induced ALI rat model

Administration of HCl to rats of the positive control group caused an increase (*p* < 0.001) in the WBCs count with decreases in RBCs count (*p* < 0.05) and in Hb levels (*p* < 0.001) when compared with the negative control group. However, treatment of ALI rats with either BM-MSCs or CoPP did not cause significant changes (*p* > 0.05) in all these hematological parameters when compared with untreated ALI rats (Table 1).

**Table 1:** Hematological parameters in different treated groups

Group	Control	ALI	ALI + BM-MSCs	ALI + CoPP
**WBCs (10**^**3**^**/μl)**	8.88 ± 1.20	13.22 ± 1.54^***^	12.51 ± 0.65^***^	11.76 ± 1.26^**^
**RBCs (10**^**6**^**/μl)**	7.88 ± 0.42	7.09 ± 0.55^*^	7.37 ± 0.07^*^	7.5 ± 0.74
**Hb (g/dl)**	12.95 ± 0.51	11.35 ± 0.32^***^	11.43 ± 0.45^***^	11.78 ± 0.60^**^

Results are expressed as mean ± SD. (*n* = 10). *ALI* acute lung injury, *WBCs* white blood cells, *RBCs* red blood cells, *Hb* hemoglobin, *BM-MSCs* bone marrow mesenchymal stem cells, *CoPP* cobalt protoporphyrin. **p < 0.01, ***p < 0.001, compared with control.

### Effects of BM-MSCs and CoPP on oxidative stress markers and TNF-α in HCl-induced ALI rat model

**Fig. 4:**
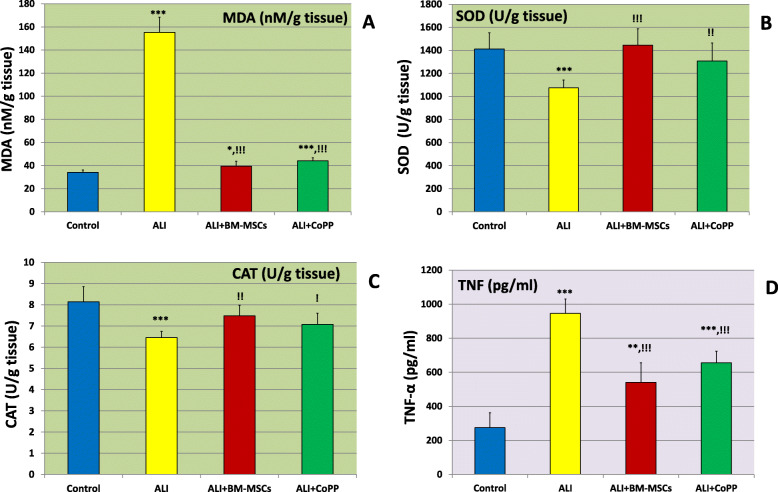
Makers of oxidative stress (**a–c**) and serum tumor necrosis factor-α (TNF-α) (**d**) in control and treated groups. Results are expressed as mean ± SD. (*n* = 10). *MDA* malondialdehyde, *SOD* superoxide dismutase, *CAT* catalase, *ALI* acute lung injury, *BM-MSCs* bone marrow mesenchymal stem cells, *CoPP* cobalt protoporphyrin. **p < 0.01 and ***p < 0.001, compared with control. !, !!, and !!!: *p* < 0.05, *p* < 0.01 and *p* < 0.001, respectively, compared with ALI

The injection of HCl to rats caused increases (*p* < 0.001) in levels of MDA (Fig. 4a) with decreases (*p* < 0.001) in the activities of SOD (Fig. 4b) and CAT (Fig. 4c) enzymes compared with the negative control group. Treatment with either BM-MSCs or CoPP to HCl-treated rats caused a significant reduction in the level of MDA and increases in SOD and CAT activities. Moreover, the BM-MSCs-treated group showed significant improvements in markers of oxidative stress than the CoPP-treated group. Also, injection of HCl to rats caused increases (*p* < 0.001) in levels of TNF-α compared with negative control rats (Fig. 4d). Treatment with either BM-MSCs or CoPP to HCl-treated rats caused a reduction in the level of TNF-α (*p* < 0.001). Moreover, the BM-MSCs-treated group showed more improvement in TNF-α than the CoPP-treated group.

### Effects of BM-MSCs and CoPP on apoptotic markers (*caspase-3* and *Bcl2*) in HCl-induced ALI rat model

**Fig. 5:**
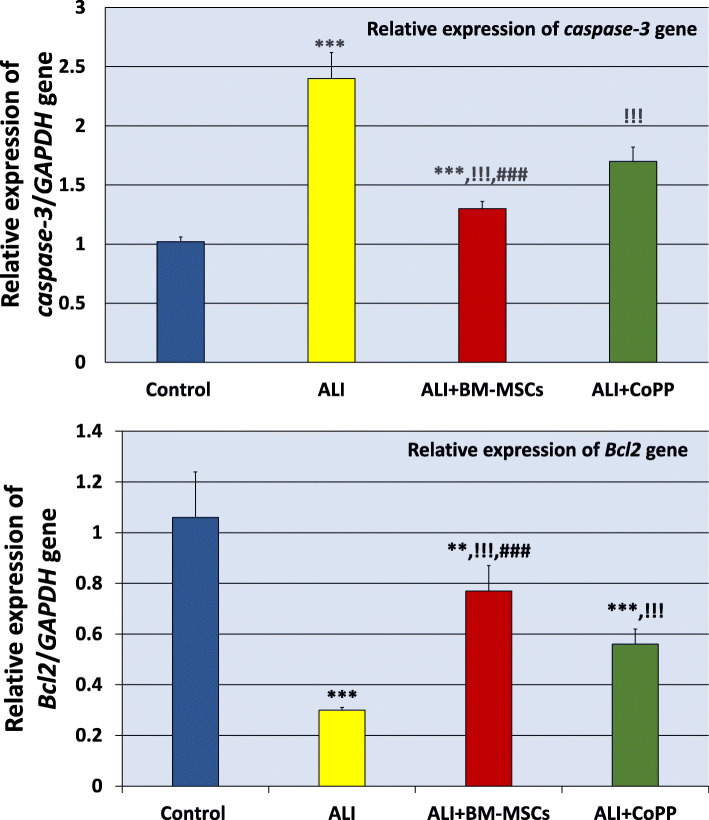
Relative expression of *caspase-3* and *Bcl2* genes in control and treated groups. Results are expressed as mean ± SD. (*n* = 10). *ALI* acute lung injury, *BM-MSCs* bone marrow mesenchymal stem cells, *CoPP* cobalt protoporphyrin. ** *p* < 0.01, ***p < 0.001, respectively, compared with control. !!!: *p* < 0.001 compared with ALI. ###: *p* < 0.001 compared with ALI + CoPP

There was upregulation (*p* < 0.001) in the expression of *caspase-3* associated with down-regulation (*p* < 0.001) in the expression of *Bcl2* in the positive control group when compared with the negative control group. This situation was reversed (*p* < 0.001) in the diseased-rats following their treatment with either BM-MSCs or CoPP compared with the untreated diseased-rats. However, by comparison, there was a noteworthy difference (*p* < 0.001) between the ALI + BM-MSCs and the ALI + CoPP groups; BM-MSCs ameliorative effect was superior to CoPP effect (Fig. 5).

### Effects of BM-MSCs and CoPP on lung morphology in HCl-induced ALI rat model

**Fig. 6:**
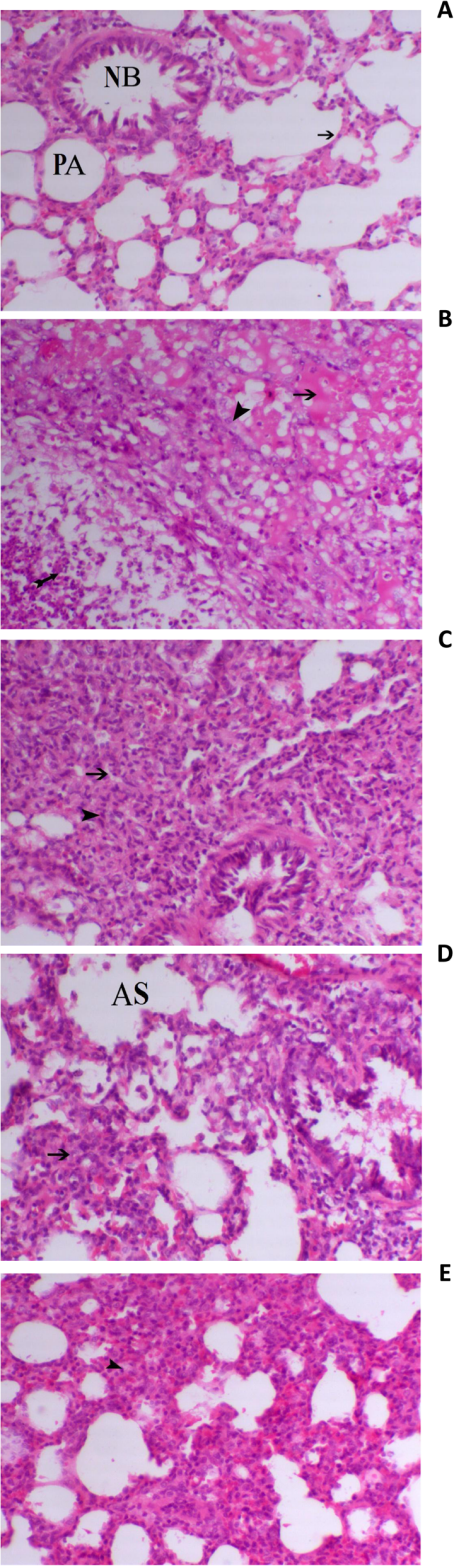
Lung specimen histopathology. **a** Normal pulmonary alveoli (PA) lined with alveolar epithelial cells (arrow) and normal bronchi (NB) lined with pseudostratified columnar cell (*normal control group*, H&E, X200), **b** epithelization of the lining epithelium of the alveoli (arrowhead), heavily serofibrinous exudation within the alveolar lumen (arrow) in addition to multifocal necrotic area filled with neutrophilic infiltration (tailed-arrow) (*ALI group*, H&E, X200), **c** complete collapse of the alveoli (arrow) associated with heavy interstitial infiltration of inflammatory cells mainly septal cells with active fibroplastic cell proliferation (arrowhead) (*ALI group*, H&E, X200), **d** decrease in interstitial tissue thickening associated with decrease septal cell proliferation (arrow) with increase in the alveolar spaces (AS), (*ALI + BM-MSCs group*, X200 H&E), and **e** moderate degree of interstitial tissue thickening (arrow) (*ALI + CoPP group*, H&E, X200)

Well-defined external lung morphology after 1 week from induction of ALI using HCl; enlarged lungs showing edema containing water, pus cells, and inflammatory cells were observed. Semi-quantitative assessment of pulmonary lesions in lung tissues of different rat groups showed great increase in necrosis with severe alveolar collapse, and severe fibrosis in the ALI group compared with the negative control rats. However, treatment with CoPP or with BM-MSCs showed good improvements in these lesions compared with the positive control. In addition, the lesions were less in the ALI + BM-MSCs group than in the ALI + CoPP group (Table 2). Figure 6a–e shows lung histopathology from different rat groups. Figure 6a depicts the normal lung architecture of the control group. Lungs of positive control animal (animal with HCl-induced ALI) showed many pathological changes including marked perivascular mononuclear cell infiltration, epithelization of the lining epithelium of the alveoli, heavily serofibrinous exudation within the alveolar lumen in addition to multifocal necrotic areas filled with neutrophilic infiltration (Fig. 6b). Evidences of complete collapse of the alveoli associated with heavy interstitial infiltration of inflammatory cells mainly septal cells with active fibroplastic cell proliferation were also obvious in the examined lung sections (Fig. 6c). Lungs of rats with HCl-induced ALI that treated with BM-MSCs showed good improvements; decreases in perivascular lymphocytic infiltration and in interstitial tissue thickening associated with decreases in septal cell proliferation with increases in the alveolar spaces (Fig. 6d). Figure 6e showed mild improvements of lung tissues of animals with HCl-induced ALI treated with CoPP; sections still show pronounced interstitial thickening and perivascular inflammatory cell infiltration.

**Table 2:** Semi-quantitative estimation of pulmonary lesions in lung tissues within the control group and in different treated groups

Group	Necrosis	Leucocytic infiltration		Alveolar collapse	Fibrosis
		Peribronchial	Interstitial		
**Control**	-	-	-	-	-
**ALI**	+++	+++	+++	+++	+++
**ALI + BM-MSCs**	+	+	+	+	+
**ALI + CoPP**	++	++	++	++	++

+++, ++, +, and - indicate severe, moderate, mild, and normal conditions, respectively.

## Discussion

The observed animal loss in the HCl-induced ALI-treated groups, in the current study, may be attributed to animal sensitivity. On contrary to our expectations, that loss was not high however it was higher in the ALI + CoPP group than in ALI + BM-MSCs suggesting the protective effect for both lines of treatment with the upper hand for BM-MSCs over CoPP. In a study on the H9N2 avian influenza virus-induced ALI, treatment by MSCs resulted in improved mice survival rate along with reductions in lung tissue injury and edema [12].

Since our hematological parameters showed a significant increase in WBCs in the ALI group, this confirmed the development of an acute pulmonary inflammatory reaction which causes migration of WBCs and damage of tissues [13]. Imam et al. [14] demonstrated a similar finding and attributed this WBCs increase to the acute inflammation. In agreement with this study and others, we found a significant increase in inflammatory cell infiltration in lung tissues by the histopathological examination of the ALI group lungs. Moreover, we found a significant increase in TNF-α level in the sera of the ALI group. It is a pro-inflammatory cytokine involved in ALI and its complicated formula ARDS [15], and it was reported in the study of Chen et al. [16] as one of the potential therapeutic targets for lung injury treatment in COVID-19 pneumonia since it showed significant increases with COVID-19 severity. The TNF-α increase is another indication of induction of the acute inflammatory reaction in lung tissues of the ALI group in our study. On the other hand, treatment with either BM-MSCs or CoPP lowered WBCs count. Previous studies demonstrated significant anti-inflammatory action for MSCs in ALI [14, 17]. The current study revealed a significant reduction in inflammatory cell infiltration in lung tissues of the BM-MSC- and CoPP-treated groups in the histopathological examination. This anti-inflammatory action probably due to inhibition of migration and infiltration of leukocytes in lung tissues or to inhibition of inflammatory cytokine secretions (e.g., TNF-α as shown in this study). Also, Seo et al. [18] reported anti-inflammatory action of CoPP by inhibiting the inflammatory mediators such as cyclooxygenase-2 and inducible nitric oxide synthase expression.

In the current study, we found significant decreases in RBCs count and Hb content in the ALI group suggesting a reduction of RBCs production by the induction of lung injury due to severe hypoxemia. Thus, the observed decreases in RBCs count and Hb content here may be explained by a suppressive effect on bone marrow erythropoiesis [19] that is potentially due to impairment in iron regulation due to inflammation. Besides, free radical intermediates may further speed up RBCs damage and disturb the erythropoiesis process. On the other hand, treatment with either BM-MSCs or CoPP resulted in significant increments in RBCs and Hb content. These increments could be explained by BM-MSC/CoPP anti-inflammatory and antioxidant impact as well as upregulation of heme oxygenase enzyme that may together improve RBCs production. The relation between heme oxygenase and MSCs will be explained later on.

Reactive oxygen species (ROS) and oxidative stress have an important role in the development and pathogenesis of a huge number of diseases and disorders including ALI [20, 21]. In the current study, we found that ALI was associated with reduced levels of SOD and CAT along with increased level of the marker of lipid peroxidation (MDA) in lung tissues suggesting enhanced oxidative stress. In agreement with these findings, previous studies reported significant decreases in the antioxidant parameters with an increase in lipid peroxidation (MDA) in induced lung injury [22]. On the other hand, treatment with either BM-MSCs or CoPP was linked to a reduction in MDA level with significant increases in the activities of SOD and CAT suggesting antioxidant activities of stem cells and CoPP. These findings might be due to the attenuation of oxidative stress by scavenging of ROS or enhancing the activity of antioxidant enzymes such as SOD and CAT by CoPP or BM-MSCs [23, 24]. Furthermore, MSCs were reported to improve the level of total antioxidant capacity [25]. This gives emphasis to the relevance of restoring antioxidant levels in the effort to safeguard the most vulnerable people from severe symptoms. In a previous study, we investigated the expression of heme oxygenase-1 (HO-1) which is induced by CoPP, and it is a key player in the cellular antioxidant system in HCl-induced lung injury leading to a decrease in inflammation and in ROS production [6, 11]. We demonstrated that the induced lung injury caused significant reduction in the expression of HO-1 at the level of mRNA, and it was upregulated by MSCs. Therefore, we can hypothesize that upregulation of HO-1 by CoPP and MSCs might be an indirect mechanism, which could explain the protective action of BM-MSCs or CoPP against ALI. This is in consistence with Zhang et al. [26].

Apoptosis or programmed cell death is another mechanism, which is involved in the pathogenesis of the induced lung injury. The regulatory proteins of apoptosis include anti-apoptotic protein (*Bcl2*) and apoptotic proteins (*caspase-3*) [27]. In the current study, we found an upregulation of *caspase-3* with downregulation of *Bcl2* in lung tissues at the levels of mRNA suggesting activation of the apoptotic process in the induced lung injury. By treatment with either CoPP or BM-MSCs, expression of *Bcl2* was upregulated and that of *caspase-3* was downregulated. However, BM-MSCs’ influence on *caspase-3* and *Bcl2* expressions was more superior. The detected high decrease in *caspase-3* by BM-MSCs might be due to their ability to secret growth factors such as hepatocyte growth factor (HGF). The latter can stimulate angiogenesis and impair apoptosis via enhancement of *Bcl2* expression and depressing of *caspase-3* expression [28]. Other studies also established that MSCs were able to decrease the lung injury via the downregulation of *caspase-3* expression [29] leading to the protection of alveolar epithelial cells from apoptosis.

As presented, our study findings highlight BM-MSCs as a potential therapy for lung injury treatment.

## Conclusions

Upon the present work results, it can be concluded that treatment of ALI with BM-MSCs or CoPP resulted in significant reductions in lung tissue damage score, MDA, *caspase-3*, and serum TNF-α levels with significant elevations in *Bcl2*, SOD, CAT, and Hb contents. The present study suggests that BM-MSCs and CoPP could attenuate HCl-induced ALI via modulation of inflammation, oxidative stress, and apoptosis. BM-MSCs curative action was more effective than that of CoPP. Therefore, BM-MSCs may be considered as one of the potent alternative treatment strategies for HCl- induced ALI.

## Data Availability

All data are included in this published article.

## Abbreviations

BM-MSCs: Bone marrow mesenchymal stem/stromal cells
CoPP: Cobalt protoporphyrin
ALI: Acute lung injury
HCl: Hydrochloric acid
WBCs: White blood cells
RBCs: Red blood cells
Hb: Hemoglobin
TNF-α: Tumor necrosis factor-alpha
MDA: Malondialdehyde
SOD: Superoxide dismutase
CAT: Catalase
ARDS: Acute respiratory distress syndrome
COVID-19: Coronavirus disease 2019
ICU: Intensive care unit
HO-1: Heme oxygenase-1
CO: Carbon monoxide
DMEM: Dulbecco's modified Eagles medium
FBS: Fetal bovine serum
RT-PCR: Real-time-PCR
SD: Standard deviation
ROS: Reactive oxygen species
